# Optimizing the interprofessional workforce for centralized intake of patients with osteoarthritis and rheumatoid disease: case study

**DOI:** 10.1186/s12960-015-0033-3

**Published:** 2015-05-28

**Authors:** Esther Suter, Arden Birney, Paola Charland, Renee Misfeldt, Stephen Weiss, Jane Squire Howden, Jennifer Hendricks, Theresa Lupton, Deborah Marshall

**Affiliations:** Workforce Research & Evaluation, Alberta Health Services, 10301 Southport Lane SW, T2W 1S7 Calgary, Alberta Canada; Workforce Research & Evaluation, Alberta Health Services, 2nd floor, Collin Barrows Building 3942 50A Avenue, PO Box 5030, T4N 6H2 Red Deer, Alberta Canada; Alberta Bone and Joint Health Institute, 400, 3280 Hospital Drive NW, T2N 4Z6 Calgary, Alberta Canada; Edmonton Musculoskeletal Centre, Suite 2068, 9499 137 Avenue NW, T5E 5R8 Edmonton, Alberta Canada; The Alberta Hip and Knee Clinic, Gulf Canada Square, Suite 335, 401- 9th Avenue, SW, T2P 3C5 Calgary, Alberta Canada; Division of Rheumatology, Alberta Health Services, Richmond Road Diagnostic & Treatment Centre, 1820 Richmond Road SW, T2T 5C7 Calgary, Alberta Canada; Alberta Bone and Joint Health Institute and Department of Community Health Sciences and Department of Medicine, Cumming School of Medicine, University of Calgary, TRW Building, 3rd Floor, 3280 Hospital Drive NW, T2N 4Z6 Calgary, Alberta Canada

**Keywords:** Access, Arthritis, Case study, Centralized intake, Interprofessional, Osteoarthritis, Rheumatoid disease, Scope of practice, Workforce planning

## Abstract

**Introduction:**

This case study was part of a larger programme of research in Alberta that aims to develop an evidence-based model to optimize centralized intake province-wide to improve access to care. A centralized intake model places all referred patients on waiting lists based on severity and then directs them to the most appropriate provider or service. Our research focused on an in-depth assessment of two well-established models currently in place in Alberta to 1) enhance our understanding of the roles and responsibilities of staff in current intake processes, 2) identify workforce issues and opportunities within the current models, and 3) inform the potential use of alternative providers in the proposed centralized intake model.

**Case description:**

Our case study included two centralized intake models in Alberta associated with three clinics. One model involved one clinic that focuses on rheumatoid disease. The other model involved two clinics that focus on osteoarthritis. We completed a document review and interviews with managers and staff from both models. Finally, we reviewed the scope of practice regulations for a range of health-care providers to examine their suitability to contribute to the centralized intake process of osteoarthritis and rheumatoid disease.

**Discussion and evaluation:**

Interview findings from both models suggested a need for an electronic medical record and eReferral system to improve the efficiency of the current process and reduce staff workload. Staff interviewed also spoke of the need to have a permanent musculoskeletal screener available to streamline the intake process for osteoarthritis patients. Both models relied on registered nurses, medical office assistants, and physicians throughout their intake process. Our scope of practice review revealed that several providers have the competencies to screen, assess, and provide case management at different junctures in the centralized intake of patients with osteoarthritis and rheumatoid disease.

**Conclusions:**

Using a broader range of providers in the centralized intake of osteoarthritis and rheumatoid disease has the potential to improve access and care specifically related to the assessment and management of patients. This may enhance the patient care experience and address current access issues.

## Background

Arthritis is a chronic disease that affects more than 4.6 million Canadians; typical symptoms of arthritis include joint pain, stiffness, and swelling [1]. If not assessed and treated early, arthritis can have debilitating effects on an individual’s physical health and overall quality of life. Arthritis is one of the leading causes of workplace disability in Canada [2, 3]. The personal and socioeconomic burden of the disease is significant [3, 4].

This case study of two centralized intake models was conducted as part of a programme of research that aims to enhance timely access to appropriate and effective treatment for patients with arthritis in Alberta. Osteoarthritis (OA) is the most common type of arthritis and is a degenerative joint disease. There is no cure for OA; however, depending on the disease severity, either a non-surgical or a surgical treatment approach could be recommended by the care provider [1]. In an end-stage disease severity, a joint replacement surgery is performed by an orthopaedic surgeon – which is often a successful treatment [5]. Rheumatoid arthritis (RA) is a chronic inflammatory autoimmune disease that requires monitoring by a specialist physician, a rheumatologist [1]. Once diagnosed with RA, patients may require treatment over their lifetime and be managed by a care provider on a regular basis to monitor disease activity [1].

There are distinct challenges for access to care for both RA and OA patients. A current and impending shortage of rheumatologists limits timely access to disease management for patients with RA [6]. The Canadian Council of Academic Rheumatologists predicted that Canada would require an increase in rheumatologists of 64 % by the year 2026 if the target of 1.9 rheumatologists per 100 000 population is to be met [7].

Similar access issues exist for OA patients. The prevalence of OA has increased in Canada over the last decade, primarily due to the ageing of the population and an increase in obesity [8, 9]. This has translated into a greater demand for orthopaedic services especially hip and knee joint replacement surgery [10]. Coupled with a limited supply of orthopaedic surgeons in Canada, the increase in demand results in long wait times for elective surgery [8, 10].

Wait times can be further increased by inappropriate referrals to specialists. For instance, patients with OA are only sometimes referred by their primary care physician to a rheumatologist first and subsequently re-referred to an orthopaedic specialist, which increases wait times for RA patients [11]. Patients with OA may also face longer wait times if appropriate screening and triage for conservative management versus surgery is not in place and non-surgical patients are referred to a surgical consult [12].

The care of stable rheumatoid or non-surgical OA patients can potentially be managed by providers other than specialists. Nurse practitioners, physiotherapists, and occupational therapists have the potential to expand their roles in the treatment of stable or non-surgical patients [13]. For example, a physiotherapist-led clinic has been explored as a means of providing care for patients who are unlikely to require surgery in the near future and for whom comprehensive conservative management may be a more appropriate option [14].

While early recognition for both OA and RA can prevent or minimize permanent and irreparable joint damage and subsequent functional impairment, the course of treatment differs for RA and OA [15, 16]. OA is a degenerative disease and typically progresses more slowly. Successful treatment of severe OA often involves hip or knee joint replacement surgery. On the other hand, early recognition of RA is critical to treatment success [1, 17]. Delays of more than 12 weeks between symptom onset and therapy initiation for RA patients may result in lower rates of remission [18]. The clinical distinction between OA and RA is an important consideration for care pathways, optimal provider involvement, and staffing models.

Centralized intake models have the potential of streamlining the intake process for various musculoskeletal disorders, including OA and RA. A centralized intake model places all referred patients on waiting lists based on severity and then directs them to the most appropriate provider or service for care [19]. In the UK, a centralized intake model was developed to provide appropriate services for musculoskeletal patients, reduce duplicate referrals, and improve waiting times [20]. This model offered a centralized point for the receipt of all musculoskeletal referrals along with a central clinical triage process that allowed patients to access the most appropriate service to meet their needs. Upon implementation, the clinic had an increase in the number of referrals. However, wait times were reduced, the number of duplicate referrals was minimized, and patients were satisfied with the service [20]. Centralized intake models for both rheumatology and OA were also introduced in Alberta to streamline the process, eliminate bottlenecks, and reduce wait times for seeing specialists [21, 22]. However, while gains were made in streamlining the process, there are barriers to care that remain for both OA and RA patients.

The focus on OA and RA as conditions is important, as these conditions consume a large proportion of specialist time. Specialists such as orthopaedic surgeons and rheumatologists also see other patients, and a patient’s condition may not yet be diagnosed before being referred to the intake clinic. As such, there is a need for triage, screening, and assessment to delineate the disease and urgency. It is necessary to review the roles and responsibilities of the health professionals currently involved in centralized intake processes to determine whether they are in fact the most appropriate and relevant provider to deliver intake services or whether there are alternative providers that may fill these roles to improve quality and gain efficiencies. Exploring the use of alternative providers in the centralized intake process may alleviate barriers to care related to the shortage of rheumatologists and orthopaedic surgeons.

This research project supports a collaborative programme of research in partnership with the Bone and Joint Health Strategic Clinical Network (BJHSCN), the Alberta Bone and Joint Health Institute, the University of Calgary, and the University of Alberta. This programme of research aims to address the need for timely access to appropriate and effective treatment for patients with OA and rheumatoid disease (RD) by developing, implementing, and evaluating an evidence-based model to optimize centralized intake, triage, and referral province-wide. The intended outcome of the new intake model is to enhance early access for OA and RD patients to comprehensive multidisciplinary assessment, triage, and referral to the most appropriate care path.

Our research focused on an in-depth assessment of two well-established centralized intake models (associated with three clinics) currently in place in Alberta. The purpose was threefold: 1) to enhance our understanding of the roles and responsibilities of health-care providers and support staff in current intake processes, 2) to identify workforce issues and opportunities within the current models, and 3) to inform the potential use of alternative providers in the above proposed centralized intake model to improve access and reduce wait times. More specifically, our research questions were as follows:

1. What are the current intake processes of existing models?
2. What type of health-care providers, physicians, and support staff are involved in the current centralized intake process?
3. What are their detailed roles and responsibilities?
4. What key competencies are required to execute these roles and responsibilities?
5. Are there other providers that could execute these roles?

### Case description

We used a case study approach to examine the intake process, the current workforce complement, and any other potential providers that may enhance access to surgical intervention and/or treatment options. Case studies allow researchers to explore multiple facets of a phenomenon rather than looking at the issue through one lens. Close collaboration between the researcher and the participant is essential to enable participants to tell their stories [23]. A case study design is relevant when the focus of the study is to answer “how” and “why” questions and researchers want to cover contextual conditions [24]. Case study research uses multiple data sources (such as documentation, interviews, and observations), which enhances data credibility [24, 25]. Finally, the report derived from case study research allows the reader to determine if the findings are relevant to their own situation.

### Sampling

Leads from the BJHSCN identified three clinics associated with two centralized intake models, an OA model (*n* = 2 clinics) and a RD model (*n* = 1 clinic) for inclusion in this study. These three clinics represent approximately 25 % of the number of centralized intake OA and RD clinics in Alberta.

### Review of clinic documents

We reviewed documents outlining the current intake and triage processes in each clinic. The documents were used to create a basic understanding of the triage process, the type of health-care providers, physicians, and support staff associated with the various steps in the process, and to inform the interviews guide. Three research team members reviewed the documents (flow charts, process maps, etc.) and extracted relevant information into a narrative describing the centralized intake process at each of the three clinics. Extracted information included process steps, any forms or documents used during the process, electronic databases or health systems used, and staff involved at each step of the process. Any discrepancies in extraction were resolved through discussion. From the documents, we created a generic referral and triage pathway that we then reviewed, refined, and validated in interviews with clinic staff.

### Interviews

We used purposive sampling to identify individuals who could speak in detail about the centralized intake process [26]. We completed the initial interviews with the managers from each clinic as they had intimate knowledge of the centralized intake process. The clinic managers facilitated access to other appropriate staff for on-site interviews. In one of the clinics, the manager also filled the role of the nurse thereby providing perspectives from both a manager and provider. Additionally, we interviewed one physician, two nurses, one administrative assistant, two case managers, one medical office assistant, and one physiotherapist for a total of 11 interviews.

At the beginning of the manager interviews, we presented them with a summary outlining the current centralized intake processes at their respective clinic. This summary was informed by the document review. We asked the managers to highlight any gaps or misrepresentations. Managers were then queried about more detailed steps in the process, staff roles and responsibilities, the challenges they experienced, and their solutions. Interviews with other staff (i.e. nurses, medical office assistants, physicians) focused on their professional background, their role in the process, knowledge and skills required to fulfil their role, and challenges with the current processes. We used a semi-structured interview guide. Data saturation was reached at the point when all interview participants reiterated the process and workforce issues [27].

### Analysis

Interviews were audio recorded to ensure accuracy and to facilitate analysis. The research team drafted a written narrative of each interview by repeatedly listening to the audio recordings. These narratives were then analysed using a general inductive approach [28] where the focus is on identifying the themes or categories most relevant to the research objectives. To enhance the rigour of our study, each research team member first independently reviewed one of the narratives to identify themes. Each team member’s analysis was reviewed and compared with each other to determine whether our interpretations of the data collected were similar. We resolved the few discrepancies in interpretation by consensus.

We sent a summary of our analysis, which included the clinic intake processes, identified issues related to the intake process, potential workforce issues, and the role of current providers and alternative providers to each of the clinic managers for external validation. Their feedback was integrated into the final findings.

### Ethics

The research project was granted ethics approval by the Conjoint Health Research Ethics Board from the University of Calgary (Ethics ID: REB13-0822). All interview participants gave written informed consent.

### Review of scope of practice regulations

We obtained publicly accessible scope of practice regulations for a range of health-care providers from Alberta’s Health Professions Act and relevant colleges, associations, and governing bodies. The regulations were reviewed to examine the potential role that alternate providers may play in the intake, referral, and treatment of OA and RD patients.

We populated a table template with a description of the various tasks in the current centralized intake processes as well as the current providers associated with each task. Three research team members reviewed the scope of practice regulations of providers whose scope included the following:

- performing musculoskeletal assessments
- reviewing medical histories
- screening for co-morbidities
- making referrals to other providers
- providing non-surgical treatment plans
- authorization to perform the following restricted activities:
- order or apply X-rays and magnetic resonance imaging (MRIs)
- administer diagnostic imaging contrast agents
- prescribe medication (Schedule 1 drug)
- dispense, compound, provide for selling, or sell a Schedule 1 drug or Schedule 2 drug within the meaning of the Pharmacy and Drug Act
- administer biologic medication

This enabled us to determine which providers have the competencies to complete the various tasks at each stage of the overall process. We resolved any discrepancies through discussion.

### Case study findings

The following centralized intake model descriptions and summary of identified workforce issues were developed based on data collected via the document review and interviews with clinic managers and staff. More specifically, the document review helped us to develop a summary of the intake process for each model while the interviews further informed the process and identified issues within the process.

### Generic centralized intake process

Both centralized intake models establish a common pathway for all referrals and a central clinical triage of patients to the most appropriate clinical service based on information received in the referral. There is variability in that some clinics conduct triage and referral review only but no screening and assessment. Based on the document review and manager interviews, we identified the following generic steps in the centralized intake processes of all three clinics:

1. Receive referral – referrals are received via fax or electronically; referrals are entered into an electronic medical record or database
2. Review referral for completeness – any missing information is requested
3. Data entry – new patient record is created and referrals information attached
4. Triage – urgency of referral is assessed and triaged accordingly; urgent patients are scheduled for immediate care, non-urgent patients are scheduled to see a musculoskeletal (MSK) screener or assigned to an appropriate wait list

The OA clinics have two additional steps:

1. Screening – patients are screened for surgery or non-surgical treatment plans; non-surgical patients are provided with a non-surgical treatment plan (e.g. physiotherapy).
2. Assessment – surgical patients are assessed to determine readiness for surgery and scheduled for any required pre-operative consults

Due to the differences in processes between the RD and the two OA clinics, we describe the RD clinic separately from the two OA clinics where appropriate.

### Centralized intake at the rheumatoid disease clinic

The intake team in Clinic 1 is comprised of a clinic manager who is also a registered nurse, one part-time registered nurse, and a unit clerk. We interviewed the clinic manager and one other staff member. According to the documentation received and the interviews, this clinic receives all rheumatology referrals across the spectrum of rheumatic diseases (RD) and receives approximately 6000 referrals per year via fax. This clinic does not have an eReferral system and much of the triaging and filing is completed manually. The lack of an interoperable electronic medical record (EMR) and eReferral system results in incomplete referrals and the duplication of patient data entry.

A unit clerk reviews each faxed referral and enters patient, referring physician, and family physician information into the rheumatology triage database. A registered nurse reviews all referrals, beginning with urgent referrals. The nurse searches for the most recent laboratory test results, diagnostic imaging, and medication history in an online provincial repository and requests any missing information from the referring physician.

The clinic staff does not do any assessments; rather, they book patients with an appropriate specialty clinic or a nurse practitioner (NP) clinic. If a referral appears to be an early inflammatory arthritis (EIA) patient, the nurse screens the patient on the phone to confirm the nature and duration of their symptoms. If the patient meets the criteria for the EIA clinic, an appointment is booked. For the remainder of the referrals, patients are booked to either a general rheumatology clinic or a specialty clinic that focuses on specific arthritis conditions such as spondyloarthropathy, lupus, vasculitis, scleroderma, or a NP clinic. The NP completes all aspects of assessment and treatment with the exception of prescribing biologic medications and has access to a rheumatologist for consultation.

Clinic 1 faces capacity issues across all specialty clinics and general rheumatology clinics. Patients with RD require ongoing follow-up with a physician. This limits the number of available appointments for new patients.

### Centralized intake at the hip and knee osteoarthritis clinics

Clinics 2 and 3 focus on patients with hip and knee OA and use similar processes. We conducted four interviews at Clinic 2 and five interviews with Clinic 3. According to the documents reviewed and the interviews, the purpose of the OA clinics is to assess both surgical and non-surgical patients and provide them with a treatment plan. The volume of referrals, which ranged from just over 5000 to 7000 patients in 2014, presents a challenge for these clinics. The clinics receive referrals via fax, which is electronically connected to the EMR, or through the mail, which is then scanned into the EMR. At the time of the interviews, eReferral was being piloted in Clinic 2, which the staff stated improves some aspects of the referral process (e.g. a referral cannot be submitted on eReferral without an attached X-ray report). eReferral, an automated referral system, was being launched in Alberta for a limited referral base of hip and knee OA and breast and lung cancer. Various staff members (medical office assistant, referral clerk) review referrals to determine patient appropriateness and urgency and to identify any missing information.

Urgent patients are booked for an appointment with the surgeon. Non-urgent referrals are reviewed by an MSK screener (either a surgeon who no longer practises surgery or a general physician) to determine if they are a potential surgical candidate or require a non-surgical treatment plan. Patients who are potential surgical candidates see a surgeon for a definitive diagnosis. The MSK screener also suggests treatments (i.e. cortisone injections) for surgical patients to optimize patients’ functionality while waiting to see the surgeon. Non-surgical patients are referred to the clinic’s interprofessional team (i.e. nurse, dietician physiotherapist, or kinesiologist) for non-surgical treatment such as weight loss, healthy living, physiotherapy, or injections. The use of an MSK screener optimizes the use of surgeon time as surgeons only see patients who are potential surgical candidates. This reduces the backlog in the system and enhances the timely management of patients. This process also offers more timely treatment to non-surgical patients who do not need to see a surgeon. The two OA clinics differ slightly in the use of an MSK screener due to funding; one OA clinic does not consistently have an MSK screener available due to limitations with the current funding model. This limits the volume of patients screened.

### Current MSK workforce for all clinics

Table 1 gives a synopsis of the current MSK workforce in all three clinics. Clerical staff and a registered nurse complete most of the processes in the RD clinic.

**Table 1:** Centralized intake providers and potential alternative providers

	Providers involved			Potential alternative providers
Stage of process	Clinic 1: rheumatic disease (RD)	Clinic 2: hip and knee osteoarthritis (OA)	Clinic 3: hip and knee osteoarthritis (OA)	
Receiving referral	Unit clerk	Executive assistant	Medical office assistant	Optimal providers^a^ in place for this stage of the process
Review referral for completeness	Nurse with RD experience	Referral clerk	Medical office assistant	Optimal providers in place for this stage of the process
Data entry	Nurse with RD experience	Referral clerk	Medical office assistant	Optimal providers in place for this stage of the process
Triage	Nurse with RD experience	Referral clerk	Medical office assistant	Optimal providers in place for this stage of the process^b^
	Unit clerk			
Screening	N/A	Surgeon	Surgeon	OA:
	The clinic only triages referrals; patients are seen by a rheumatologist and allied health team at the rheumatology clinics.		General practice physician	Physiotherapist
				Chiropractor
				Advanced practice nurse
				RD: interprofessional MSK team
Assessment	N/A	Surgeon	Surgeon	OA:
	Assessment process out of scope. Staff would like the rheumatologist to see patients for initial diagnosis and development of a treatment plan. An interprofessional team would be responsible for case management and ongoing assessment of stable patients.	Medical office assistant	Medical office assistant	Occupational therapist
		Nurse	Nurse	Kinesiologist
		Dietician	Dietician	Chiropractor
		Kinesiologist and/or physiotherapist	Physiotherapist	RD: interprofessional MSK team
			Occupational Therapist	

^a^Optimal providers are those operating at the right level; these providers are not overqualified but have the appropriate level of knowledge and skills to complete the task

^b^Optimal providers include a nurse required for RD but referral clerk or medical office assistant for OA given the clinical distinction between RD and OA. There are over 100 different rheumatic diseases with a multitude of presenting symptoms thus making the referral review challenging

Both OA clinics use clerical staff for the initial referral review and triage and physicians for screening. Furthermore, surgeons, medical office assistants, and nurses are involved in the assessment process for surgical and non-surgical patients. However, the two OA clinics differ in the use of other providers during the assessment process. One of the OA clinics involves kinesiologists while the other OA clinic uses occupational therapists and physiotherapists in the assessment process. Staff of the three clinics identified several issues with the current intake models including the need for electronic medical records and eReferrals to reduce staff workload. They also raised several specific issues related to optimizing the interprofessional workforce.

### Identified workforce issues at the rheumatoid disease clinic

i. Bottleneck due to lack of available rheumatologists

Staff identified the lack of rheumatologists as the main contributor to the bottleneck in the system. They noted that this was always an issue for the clinic and was gradually worsening due to retirements and rheumatologists moving away. While there was a short wait time for urgent referrals, routine and stable patient wait times to see a rheumatologist had increased to over 3 months. Staff stated that an MSK screener could be beneficial, particularly for vague referrals. They argued that due to the complex nature of conditions received at the clinic, the MSK screener would need to be a highly skilled and experienced individual who could appropriately determine if patients needed to be seen by a rheumatologist.

ii Lack of MSK case-management team for stable and non-urgent patients

The RD clinic lacks an interprofessional team to provide case management and ongoing assessments of stable patients. Staff suggested that several providers (i.e. physiotherapists, nurse practitioner) could potentially see stable and non-urgent patients. While they could schedule stable patients to an NP with his own practice, he had a full caseload and was not able to accept new patients. Staff suggested that an interprofessional MSK team would be able to effectively develop and implement treatment plans and complete patient follow-up for stable patients. Non-urgent patients on the wait list have limited support. Staff stated that these patients would benefit from education as well as conservative management through an interprofessional MSK team. An MSK team would mitigate the risk of deterioration for patients on a wait list as well as reduce the number of patients that need to be seen by the rheumatologist on a regular basis following treatment. Funding would be required to resource extra staff and associated training.

### Identified workforce issues at the osteoarthritis clinics

i. Support for MSK screener

For Clinics 2 and 3, the interviewed staff noted that having sustainable support for a permanent MSK screener is a critical factor for streamlining the intake process. In Clinic 3, two orthopaedic surgeons who were no longer performing surgery and a general practitioner provided the MSK screening. This allowed the clinic to reduce the number of non-surgical patients seen by the surgeons for surgical consult. The MSK screener only screened the next available wait list, not the direct referral wait list due to capacity issues. Staff stated that it would be more effective overall for the screener to support all surgeons and all patients. The role of the screener was to determine if referrals were surgical or not. Non-surgical patients were provided a non-surgical treatment plan, whereas surgical patients were booked into a surgical consult. Staff suggested that other providers such as a physiotherapist in an advanced role could potentially fill the MSK screener role.

In Clinic 2, MSK screening was more *ad hoc* due to funding issues and was done by one of the active surgeons as time allowed. The MSK screeners billed Alberta Health as a consult, so the clinic did not directly pay for the screeners. The staff at this clinic saw a potential role for advanced practice physiotherapists or NPs to complete the screening. They stressed though that this would require revisions to the funding model to enable alternative providers to complete the screening. In the current funding model, reimbursement occurs for services delivered by surgeons or physician screeners on a case rate for each procedure (i.e. surgical patient, non-surgical patient, and MSK visit).

Despite the differences in processes observed between the three clinics, a common theme emerged. Bottlenecks existed in part because of limited availability of specialists (surgeons, rheumatologist) who currently see a majority of the patients. Staff at all three clinics suggested the use of an interprofessional MSK team for screening, assessing, and managing non-surgical OA patients and stable RD patients. This would allow better use of the specialist’s time and increase patients’ access to screening services. Consideration will need to be given to a sustainable funding model for the MSK team.

### Scope of practice review

We examined the current scope of practice for different providers in Alberta to determine which providers may be suitable for various responsibilities associated with MSK screening, triaging, and assessment (Table 2).

**Table 2:** Competencies of alternative providers for centralized intake

Responsibilities	Nurse practitioner	Registered nurse	Licensed practical nurse	Physiotherapist	Occupational therapist	Chiropractor	Therapy assistant	Athletic therapist	Kinesiologist	Recreation therapist
MSK assessment	X	X	X	X	X^a^	X	X^b^	X	X	
Assess medical history	X	X	X	X	X	X				
Screen for co-morbidities	X	X				X				
Order or apply X-rays and MRIs	X			X		X				
Non-surgical treatment plan (i.e. nutrition, lifestyle, exercise)	X	X	X	X	X	X	X	X	X	X
Case management	X	X		X		X				X
Refer to other providers	X	X		X	X^c^	X				
Administer diagnostic imaging contrast agents	X	X	X		X					
Prescribe medication (Schedule 1 drug)	X									
Dispense, compound, provide for selling, or sell a Schedule 1 drug or Schedule 2 drug within the meaning of the *Pharmacy and Drug Act*	X	X								
Administer biologic medication										

^a^Occupational therapists can conduct a functional capacity assessment which is a physical assessment of an individual’s ability to perform work-related activity

^b^Therapy assistants can assess functional mobility as part of an MSK assessment

^c^Occupational therapists can recommend appropriate resources or other service providers when the service is requested

NPs have the competencies to provide services for both RD and OA clinics. They have the most comprehensive scope and can complete a wide range of responsibilities, with the exception of administering biologic medications [29]. Other regulated providers, such registered nurses, chiropractors, and physiotherapists have a broader role to play in centralized intake clinics. The scope of registered nurses is very broad, but in contrast to NPs, they cannot order X-rays or prescribe medications. A physiotherapist, chiropractor, or occupational therapist can fulfil many of the responsibilities associated with assessment, case management, and screening [30–33]. Chiropractors can also screen for co-morbidities, a competency that is not within the scope of physiotherapists or occupational therapists [31]. All three professional groups have limitations for prescribing and dispensing medication; NPs can prescribe a limited range of medications in Alberta. Generally, the scope of unregulated providers (therapy assistant, athletic therapist, kinesiologist, and recreation therapist) is much narrower, but most of them have the ability to conduct basic MSK assessments and develop non-surgical treatment plans, such as nutrition and lifestyle counselling or exercise plans.

Based on this significant scope overlap, we re-examined the providers associated with the current processes and considered potential alternative providers for each stage of the centralized intake process to improve access (Table 1 last column). Optimal providers are those operating at the right level; these providers are not overqualified but have the appropriate level of knowledge and skills to complete the task. The optimal providers are in place for receiving and reviewing the referral, data entry, and triage. A referral clerk or medical office assistant fulfilled these roles in the OA clinics, and a unit clerk or nurse with RD experience carried out these roles in the RD clinic.

According to the clinic staff interviewed, there are potential areas where alternative providers could be positioned to improve access to services in both OA and RD clinics. A physiotherapist, advanced practice nurse, or chiropractor could potentially complete the MSK screening in the OA clinics. All three providers have a sufficiently broad scope, substantive training in MSK assessments, and the ability to refer to other providers and order X-rays [29–31].

Given the different nature of RD patients, an interprofessional MSK team with an NP or advanced practice nurse could complete MSK screening and assessment in the RD clinic. The RD clinic only triages referrals; patients are not seen for MSK screening or for assessment to determine the most appropriate care path. The number and type of providers on the interprofessional MSK team will depend on the screening and assessment process developed for the RD clinic. Physiotherapists, occupational therapists, kinesiologists, and dieticians would likely add value by developing comprehensive conservative care plans and providing patient education to help patients to be more engaged in their care.

### Discussion and evaluation

The purpose of this case study was to identify workforce issues and opportunities within two well-established centralized intake models for OA and RD and to inform the potential use of alternative providers. The existing models highlight a number of opportunities to engage a range of alternative providers to address current bottlenecks, improve timely access to care, and enhance the patient care experience and ultimately patient outcomes.

The evidence of positive outcomes associated with timely and appropriate access to care for arthritis is strong [34]. These positive outcomes include a greater chance of remission of the disease and less progressive joint damage, thus improving the likelihood of these individuals to remain in the workforce [34]. Despite this evidence, individuals with arthritis continue to face barriers that delay access to treatment. Challenges in providing timely arthritis care include a shortage of specialist providers (i.e. rheumatologists) and inefficiencies in the referral process such as high numbers of incomplete and inappropriate referrals [20, 35, 36]. Centralized intake models provide an opportunity to improve access to appropriate care for arthritis patients, create consistency in care, and better utilize human resources including the more efficient use of specialists’ time [17, 20, 37].

One such model, the Central Intake and Assessment Centres (CIAC), has been implemented in Ontario and makes use of alternative providers. CIACs were established to receive referrals for hip and knee replacement procedures. Patients undergo a comprehensive physical assessment to determine the appropriateness for surgical consult, typically by a non-surgeon assessor such as a trained physiotherapist or advanced practice nurse. A recent evaluation of CIAC has demonstrated success in streamlining the intake process for surgeons and providing patients with a choice for next available surgeon or conservative management [38]. The CIAC model has also positively affected wait time between referral and initial consultation. Both patients and surgeons report high levels of satisfaction with this model. For example, the majority of surgeons reported satisfaction with the non-surgeon assessor assessments and valued the ability to spend less time assessing patients who were not surgical candidates [38]. The hip and knee OA clinics in this study already have some of these components in place. For example, both OA clinics provide patients with a choice for next available surgeon or conservative management. In addition, one OA clinic has positively affected wait time between referral and consultation due to sustainable MSK funds.

The interview participants identified NPs and physiotherapists as having the skill sets required to conduct activities related to screening, assessment, and conservative treatment of OA and RD patients. Involving alternative providers in MSK screening would require training on appropriate screening protocols and criteria to create consistency in identifying OA patients eligible for surgery. The two OA clinics make differential use of physiotherapists, occupational therapists, and kinesiologists. It is unlikely that these differences are based on variation in patient populations; rather, it demonstrates some flexibility in deployment of human resources within the centralized intake process as all three providers have the skills required for MSK assessments.

There is some evidence supporting the involvement of alternative providers in the screening, assessment, and conservative treatment of RA to improve patient access to high-quality care. Solomon and colleagues [39] found that advanced practice NPs and physician assistants practising in rheumatology in the U.S. had broad responsibilities, which included starting patients on medications, performing intake assessments, and patient education. The majority of the NPs and physician assistants surveyed by Solomon and colleagues [39] indicated they had their own panel of patients, worked with a high level of independence, and consulted with a rheumatologist only when needed. These findings suggest that these types of providers may in fact help to expand the expertise in rheumatology thereby improving access to medical services for RD patients, building capacity in rheumatology services, and providing better patient care. Furthermore, Li and colleagues [13] also promote the expansion of roles of rehabilitation, nursing, and pharmacy professionals in rheumatology to improve access and reduce the delay from the onset of symptoms to treatment for the early stages of RA.

Not mentioned by interview participants is the possibility of using chiropractors in the centralized intake. Chiropractors are not currently involved in the OA assessments but would be well suited for this role based on their considerable scope of practice and extensive training in MSK assessments. As highlighted in Table 2, they could in fact complete many of the activities involved in managing patients and add value to an interprofessional MSK team. Some models that utilize chiropractors for assessing eligibility of MSK patients for spine surgery are being implemented in Ontario and show some preliminary positive outcomes such as reduced number of inappropriate referrals and use of diagnostic imaging [40].

### Practical implications

Given the existing shortage of specialists for the management of OA and RA patients, it is imperative that we explore the possibility of optimizing the roles of alternative providers to fill the gap and improve efficiencies within our health system [8, 10]. Current centralized intake models are making limited use of interprofessional team members such as NPs, physiotherapists, chiropractors, and occupational therapists. We suggest that better use of an interprofessional team in screening, assessment, and management of arthritis patients could potentially reduce patient wait times and result in the more appropriate use of surgeon time for OA patients as well as better availability of rheumatologists. The use of a trained physiotherapist or advanced practice nurse in the intake process of CIACs in Ontario proved successful thereby lending evidence to support the incorporation of an interprofessional team in centralized intake models [38]. Interprofessional teams in rheumatology have also been promoted in the literature as a means to provide patients’ timely and more coordinated care and increase access to providers that target the many facets of the illness such as physiotherapists, occupational therapists, pharmacists, and social workers [41, 42].

While several providers could complete activities within the centralized intake process, it is important to note that providers from different disciplines will likely bring different perspectives to the assessment or management of patients based on their professional philosophies. Their training will further equip them with different skill levels around assessment and treatment. The importance of content knowledge and experience emerged from the interviews. Interview participants at all clinics noted that extensive on-the-job training and backgrounds in rheumatology and OA are invaluable and greatly influence the enactment of their roles; new graduates would likely not be suited for these roles. When establishing a model with an interprofessional MSK team, it is therefore important to fully understand the different knowledge and skills providers bring to the team as well as potential gaps. Providers may need additional education relating to specific aspects of assessment and management that may not be part of the provider’s professional training. For example, Ontario has an Advanced Clinician Practitioner in Arthritis Care (ACPAC) programme that trains physiotherapists, occupational therapists, and nurses with the requisite skill sets to manage stable patients [43]. The CIAC model recommends the ACPAC training, in addition to individual training with surgeons for non-surgeon assessors [38]. Furthermore, it will be essential to develop a shared philosophy, role clarity, and an agreed upon care pathway so providers can work synergistically.

### Limitations

The BJHSCN identified the clinics and clinic managers based on their knowledge of the centralized intake models. This may have led to a selection bias in the MSK clinics included in this research project. Other limitations relate to case study design more generally and include the inability to generalize findings from case studies to a broader context and limitations associated with small numbers of interviews. Other centralized clinics in Alberta may have additional workforce challenges related to other factors such as geography that were not captured in this study.

## Conclusions

In summary, based on our review, we suggest that opportunities exist to enhance the existing staffing models to include a broader range of providers in particular in the screening and assessment process of centralized intake models. This might enhance the patient care experience as well as address access issues that currently exist.

## Abbreviations

ACPAC: Advanced clinician practitioner in arthritis care
BJHSCN: Bone and Joint Health Strategic Clinical Network
CIAC: Central intake and assessment centres
EIA: Early inflammatory arthritis
OA: Osteoarthritis
EMR: Electronic medical record
MSK: Musculoskeletal
NP: Nurse practitioner
RA: Eheumatoid arthritis
RD: Rheumatic disease

## References

[1] Arthritis Alliance of Canada. The impact of arthritis in Canada: today and over the next 30 years. 2011. http://www.arthritisalliance.ca/images/PDF/eng/Initiatives/20111022_2200_impact_of_arthritis.pdf. Accessed 25 Sept 2014.

[2] The Arthritis Society. Arthritis in Canada: facts and figures. 2014. http://www.arthritis.ca/document.doc?id=925. Accessed 25 Sept 2014.

[3] Kaptein S, Gignac M, Badley E (2009). Differences in the workforce experiences of women and men with arthritis disability: a population health perspective. Arthritis Rheuma (Arthritis Care Res).

[4] Li X, Gignac M, Anis A (2006). The indirect costs of arthritis resulting from unemployment, reduced performance and occupational changes while at work. Med Care.

[5] Gillam MH, Lie SA, Salter A, Furnes O, Graves SE, Havelin LI (2013). The progression of end-stage osteoarthritis: analysis of data from the Australian and Norwegian joint replacement registries using a multi-state model. Osteoarthritis and Cartilage.

[6] Vanderby SA, Carter MW, Noseworthy T, Marshall DA (2015). Modeling the complete continuum of care using system dynamics: the case of osteoarthritis in Alberta. J Simul.

[7] Hanly J (2001). Manpower in Canadian academic rheumatology units: current status and future trends. J Rheumatol.

[8] Badley EM, Davis AM (2012). Meeting the challenge of the ageing of the population: issues in access to specialist care for arthritis. Best Practice Res Clin Rheumatol.

[9] Comeau P (2004). Crisis in orthopedic care: surgeon and resource shortage. Can Med Assoc J.

[10] Badley EM, Canizares M, Mahomed N, Veinot P, Davis AM (2011). Provision of orthopaedic workforce and implications for access to orthopaedic services in Ontario. J Bone Joint Surg.

[11] Marshall D, Enns E, Vanderby S, Frank C, Wasylak T, Mosher D (2013). Estimating incidence and prevalence of osteoarthritis (OA) in Alberta using administrative claims data. Value Health.

[12] Hawker G, Bohm E, Connor-Spady B, De Coster C, Dunbar M, Hennigar A, et al. Perspectives of Canadian stakeholders on criteria for appropriateness for total joint arthroplasty in patients with hip and knee osteoarthritis. Arthritis & Rheumatology. 2015; In press. 10.1002/art.39124 25930243

[13] Li LC, Badley EM, MacKay C, Mosher D, Jamal S, Jones A (2008). An evidence-informed, integrated framework for rheumatoid arthritis care. Arthritis Rheuma.

[14] Doerr C, Graves S, Mercer G, Osborne R (2013). Implementation of a quality care management system for patients with arthritis of the hip and knee. Aust Health Rev.

[15] Chao J, Kalunian K (2010). Managing osteoarthritis. A multidisciplinary approach. J Musculoskelet Med.

[16] Choy E, Scott D, Kingsley G, Williams P, Wojtulewski J, Papasavvas G (2002). Treating rheumatoid arthritis early with disease modifying drugs reduces joint damage: a randomised double blind trial of sulphasalzaine vs diclofenac sodium. Clin Exp Rheumatol.

[17] Villaneuve E, Nam JL, Bell MJ, Deighton CM, Felson DT, Hazes JM (2014). A systematic literature review of strategies promoting early referral and reducing delays in the diagnosis and management of inflammatory arthritis. Ann Rheum Dis.

[18] Norton S, Koduri G, Nikiphorou E, Dixey J, Williams P, Young A (2013). A study of baseline prevalence and cumulative incidence of comorbidity and extra-articular manifestations in RA and their impact on outcome. Rheumatology.

[19] Marshall DA, Burgos-Liz L, Maarten JI, Osgood ND, Padula WV, Higashi MK (2015). Applying dynamic simulation modeling methods in health care delivery research – the SIMULATE checklist: report of the ISPOR simulation modeling emerging good practices task force. Value Health.

[20] Maddison P, Jones J, Breslin A, Barton C, Fleur J, Lewis R (2004). Improved access and targeting of musculoskeletal services in northwest Wales: targeted early access to musculoskeletal services (TEAMS) programme. Br Med J.

[21] Bichel A, Erfle S, Wiebe V, Axelrod D, Conly J (2009). Improving patient access to medical services: preventing the patient from being lost in translation. Healthc Quar.

[22] Frank C, Marshal D, Faris P, Smith C (2011). Essay for the CIHR/CMAJ Award: improving access to hip and knee replacement and its quality by adopting a new model of care in Alberta. Can Med Assoc J.

[23] Crabtree BF, Miller WL, Crabtree BF, Miller WL (1999). Researching practice settings: a case study approach. Doing qualitative research.

[24] Yin RK (2006). The case study anthology.

[25] Patton MQ (1990). Qualitative evaluation and research methods.

[26] Tongco D (2007). Purposive sampling as a tool for informant selection. Ethnobotany Res Appl.

[27] Crabtree BF, Miller WL, Crabtree BF, Miller WL (1999). Sampling in qualitative inquiry. Doing qualitative research.

[28] Thomas D (2006). A general inductive approach for analyzing qualitative evaluation data. Am J Eval.

[29] College & Association of Registered Nurses of Alberta. Nurse practitioner (NP) competencies. 2011. Accessed 1 Jul 2014. http://www.nurses.ab.ca/content/dam/carna/pdfs/DocumentList/Standards/NP_Competencies_Jan2011.pdf.

[30] Physiotherapy Alberta College & Association. Standards of practice for Alberta physiotherapists. 2012. Accessed 1 Jul 2014. http://www.physiotherapyalberta.ca/files/practice_standards_all_2012_revised.pdf.

[31] Alberta College and Association of Chiropractors. Standards of practice. 2014. Accessed 1 Jul 2014. http://www.albertachiro.com/ieadmin/files/ACAC_Standards_of_Practice.pdf.

[32] Alberta College of Occupational Therapists. Standards of practice. 2003. Accessed 1 Jul 2014. http://www.acot.ca/files/Standards_of_Practice.pdf.

[33] Alberta College of Occupational Therapists. Essential competencies of practice for occupational therapists in Canada. 2003. Accessed 1 Jul 2014. http://www.acot.ca/files/Essential_Competencies_of_Practice.pdf.

[34] Arthritis Alliance of Canada. A pan-Canadian approach to inflammatory arthritis models of care. 2014. Accessed 9 Oct 2014. http://www.arthritisalliance.ca/images/PDF/eng/20140430-2030-IA-MOCFINAL.pdf.

[35] Kur J, Koehler B (2011). Rheumatologist demographics in British Columbia: a looming crisis. Br Columbia Med J.

[36] Speed CA, Crisp AJ (2005). Referrals to hospital-based rheumatology and orthopaedic services: seeking direction. Rheumatology.

[37] MacKay C, Veinot P, Badley E (2008). Characteristics of evolving models of care for arthritis: a key informant study. BMC Health Serv Res.

[38] Deloitte and Touche LLP. Evaluation of central intake and assessment centres for hip and knee replacement: final report. 2011. Accessed 16 Jul 2014. http://www.google.ca/url?sa=t&rct=j&q=&esrc=s&source=web&cd=1&ved=0CB0QFjAA&url=http%3A%2F%2Fwww.southeastlhin.on.ca%2F~%2Fmedia%2Fsites%2Fse%2FUploadedFiles%2FHSP%2FProjectsInitiatives%2F6%2520-%2520CIAC%2520Hip%2520and%2520Knee%2520Final%2520Report%2520Exec%2520Summ.pdf&ei=OGRnVby9CJbqoATJ5oAY&usg=AFQjCNGVDrxs-lwG3U8_r2nlbRZkLUEzyw.

[39] Solomon DH, Bitton A, Fraenkel L, Brown E, Tsao P, Katz JN (2014). Roles of nurse practitioners and physicians assistants in rheumatology practices in the US. Arthritis Care Res.

[40] Ontario Chiropractic Association. Innovative models of care in Ontario. 2013. Accessed 9 Oct 2014. https://d2oovpv43hgkeu.cloudfront.net/Collaboration/Chiropractic-Collaboration-Innovative-Models-of-Care-in-Ontario.pdf.

[41] Marion C, Balfe L (2011). Potential advantages in interprofessional care in rheumatoid arthritis. J Manag Care Pharm.

[42] Walker J (2012). Rheumatoid arthritis: role of the nurse and multidisciplinary team. Br J Nurs.

[43] Lundon K, Shupak R, Reeves S, Schneider R, McIlroy JH (2009). The advanced clinician practitioner in arthritis care program: an interprofessional model for transfer of knowledge for advanced practice practitioners. J Interprof Care.

